# ABA signaling converts stem cell fate by substantiating a tradeoff between cell polarity, growth and cell cycle progression and abiotic stress responses in the moss *Physcomitrium patens*

**DOI:** 10.3389/fpls.2023.1303195

**Published:** 2023-11-29

**Authors:** Marcel Pascal Beier, Chiyo Jinno, Natsumi Noda, Kohei Nakamura, Sumio Sugano, Yutaka Suzuki, Tomomichi Fujita

**Affiliations:** ^1^Department of Biological Sciences, Faculty of Science, Hokkaido University, Sapporo, Hokkaido, Japan; ^2^Institute for the Advancement of Higher Education, Hokkaido University, Sapporo, Hokkaido, Japan; ^3^Division of Life Science, Graduate School of Life Science, Hokkaido University, Sapporo, Hokkaido, Japan; ^4^Department of Computational Biology and Medical Sciences, Graduate School of Frontier Sciences, The University of Tokyo, Kashiwa, Chiba, Japan

**Keywords:** abscisic acid (ABA), cell cycle, cell division, cell polarity, Physcomitrium patens, stress response

## Abstract

Abscisic acid (ABA)-mediated abiotic stress tolerance causes plant growth inhibition. Under such stress conditions, some mosses generate *de novo* stress-resistant stem cells, also called brood cells or brachycytes, that do not exist under normal conditions. However, the cell physiological basis of the growth inhibition and the stem cell formation is not well understood. Here, we show that the ABA-induced growth inhibition of the moss *Physcomitrium patens* apical protonemal cells (protonemal stem cells) is mediated through a shift from asymmetric to symmetric cell division. This change of the cell division mode, and consequently change of stem cell activity, is substantiated by dampening cell polarity and cell proliferative activity through the altered distribution of cytoskeletal elements, the mitotic spindle and the vacuole, which results in the production of stress-resistant stem cells. Alteration of the cell physiological data is supported by the results of RNAseq analysis indicating rapid changes in both cell polarity and cell cycle regulation, while long-term treatments with ABA for 5 to 10 days impact mainly the transcriptional and translational regulation. The regulation of cell polarity and cell cycle genes suggests growth arrest mediated by small GTPases (ROPs) and their guanine exchange factors (ROPGEFs) and by cyclin and cyclin-dependent-kinase complex, respectively. Our data suggest that a tradeoff relationship between growth ability and abiotic stress response in the moss is substantiated by ABA signaling to suppress cell polarity and asymmetric cell growth and may play a pivotal role in stem cell fate conversion to newly produced stress-resistant stem cells.

## Introduction

Abiotic stresses such as salinity and drought have a negative impact on plant growth and productivity worldwide. The ability to withstand unfavorable conditions often goes hand in hand with growth suppression, which is known as a tradeoff between growth and stress response in plants (Zhang et al., 2020; Chen et al., 2021; Dwivedi et al., 2021). Abscisic acid (ABA), a plant hormone and a well-known player in the defense against abiotic stress, drastically limits plant growth. One of the underlying signaling pathways goes through core components of ABA signaling, PYR/PYL/RCAR ABA receptors, PP2C phosphatases, and SnRK2, and with calcium-dependent kinases (CPKs), which regulate guanine exchange factors (ROPGEFs) that modulate small GTPases (ROPs) (Ma et al., 2009; Park et al., 2009; Cutler et al., 2010; Raghavendra et al., 2010; Joshi-Saha et al., 2011; Li et al., 2018). The regulation of ROPs by ROPGEFs modulates cellular processes such as polarized cell growth and cell division that are all linked to asymmetric cell growth (Humphries et al., 2011; Oda & Fukuda, 2012; Yang & Lavagi, 2012). While this signaling pathway underscores a significant role of ABA signaling that limits plant growth in response to abiotic stress, the entire molecular mechanism for control of the tradeoff and cell physiological changes that contribute to the growth inhibition must be more complex than is currently understood and therefore needs to be further explored (Zhang et al., 2010; Nonogaki et al., 2020; Hasan et al., 2022; Li et al., 2022). Since plant stem cells determine the general cell population size and therefore influence plant growth, analysis and an understanding of stem cells would be promising for this purpose. While it is difficult to observe stem cells in most flowering plants due to their presence in complex multicellular meristems, easily accessible stem cells are present in the moss *Physcomitrium patens* (formerly *Physcomitrella patens*) and are in the forms of apical protonema cells, chloronemal apical stem cells and caulonemal apical stem cells (Kofuji & Hasebe, 2014). These cells exhibit tip growth and undergo asymmetric cell division, which is a typical stem cell feature (Rounds & Bezanilla, 2013; Yi & Goshima, 2020). In contrast, abiotic stresses, such as low temperature, drought and salt stress, and treatment with exogenous ABA suppress protonemal cell growth and convert their cell fate to brood cells, also called brachycytes or diaspores, which are stress-resistant cells. The appearance of brood cells suggests that a tradeoff between growth and stress response exists in bryophytes as well as in flowering plants and has been their important adaptive strategy against harsh terrestrial environments (Schnepf & Reinhard, 1997; Decker et al., 2006; Arif et al., 2019).

In a previous study we demonstrated that application of ABA to *P. patens* protonema switches a cell division mode from asymmetry to symmetry, which indicates a loss of the apical stem cell features under the condition of prolonged ABA treatment, leading to the formation of stress-resistant brood cells (Hiroguchi et al., 2022). In the presence of ABA, for example, for more than one week, brood cells continue to proliferate through symmetric cell division, producing two daughter cells of equal nature, both of which are stress-resistant. In contrast, the removal of ABA or the absence of stress causes them to regenerate normal apical cells through asymmetric cell division (Hiroguchi et al., 2022). Thus, the brood cell is a type of stem cell that newly emerges under stress conditions; brood cells perform symmetric cell division to self-renew under stress conditions and asymmetric cell division to produce normal apical stem cells when stress is removed (Hiroguchi et al., 2022). Studying the process of how brood cells emerge in response to stress might enable elucidation of the molecular mechanism of the tradeoff between plant cell growth and stress resistance. Several reports have shown changes in global gene expression after ABA treatment (maximum periods of 2 to 12 hours); although activation of the ABA signaling cascade and its downstream genes involved in stress response have been revealed, indicating conservation of stress response between *P. patens* and *Arabidopsis thaliana*, the genes that inhibit growth have not been investigated in detail (Cuming et al., 2007; Richardt et al., 2009; Komatsu et al., 2013; Khraiwesh et al., 2015; Stevenson et al., 2016; Zhao et al., 2018; Arif et al., 2019; Shinozawa et al., 2019) Also, the cellular physiological changes in protonemal apical stem cells towards growth inhibition are poorly understood.

In this study, we analyzed the cellular physiological basis of this phenomenon further through a time course analysis of apical cell growth. We were able to confirm that the core ABA module through the action of SnRK2 played a pivotal role in growth inhibition, while one of the downstream transcription factors of ABA signaling, ABA-insensitive 3 (ABI3), did not play a pivotal role. The decrease in apical cell growth was substantiated through changes in the cytoskeleton, the mitotic spindle formation and the vacuole, that all indicated a shift from asymmetric to symmetric cell division. RNAseq analysis of the time course of ABA treatment indicated major changes in organelle-related gene groups in the initial two days, while long-term exposure, up to 10 days, led to changes in transcriptional and translational regulation. Further analysis of cell cycle-related genes indicated fast and long-lasting effects of ABA treatment. Taken together, our work reveals a tradeoff between growth, which is maintained in apical stem cells through asymmetric cell division, and the ABA induced-stress response, which disturbs the physiological basis of asymmetric division for higher stress resistance, resulting in *de novo* formation of stress-resistant stem cells.

## Materials and methods

### Plant materials and growth conditions

*P. patens* (Hedw.) Bruch & Schimp ssp. patens Tan strain was used as the wild type in this study (Nishiyama et al., 2000). *ProEF1α:D2* (as indicated by WT), *ProEF1α:D2/ppsnrk2qko* (*snrk2qko*) and *ProEF1α:D2/ppabi3tko* (*abi3tko*) lines were described previously (Kitagawa & Fujita, 2013; Tomoi et al., 2020). The plants were cultured on BCDAT medium with 0.8% (w/v) agar (Nacalai Tesque) under continuous white light at 25°C (Nishiyama et al., 2000). GFP-Tubulin (Hiwatashi et al., 2008) and LifeAct-Venus (Era et al., 2009) were used as cytoskeleton markers. GFP-AtVam3 was used as a vacuole marker (Oda et al., 2009). For all experiments, protonema cells were embedded in 800 µL BCD with 0.8% (w/v) agar or BCDATG with 0.5% (w/v) gellan gum (Wako, Osaka, Japan) instead of agar in 35 mm Petri dishes with a 27-mm coverslip window at the bottom (Iwaki, Japan). After inoculation, protonemal cells were cultured for 7 days under white light. To apply ABA to protonemal cells for live imaging, BCDATG liquid medium containing ABA (500 µM) or 1% (v/v) DMSO was added so that the final concentrations were 50 µM and 0.1%, respectively.

### Microscopy

Tip growth and cell cycle progression were observed with an inverted microscope (ECLIPSE Ti-E, Nikon). Cytoskeleton markers and a vacuole marker were observed with an inverted microscope (ECLIPSE Ti2, Nikon) equipped with a spinning disk head (X light V3, CrestOptics) with a 1.40 NA 60× oil immersion objective (Plan Apo VC 60× Oil, Nikon) at room temperature. Illumination with 470-nm and 520-nm laser light was used for GFP and Venus excitation, respectively. Emission filters were 510/50 nm for GFP and 560/40 nm for Venus. Image acquisition was controlled by MetaMorph (Molecular Devices). All images were processed using Fiji.

### RNA sequencing

Total RNA from 50 µM ABA-treated protonemata tissues was extracted using the RNeasy Plant Mini Kit (Qiagen). Tissues were sampled after 0, 1, 3, 8, 24, 48, 120 and 240 hours. No technical replicates were sampled for the different time points. After RNA quality check, the high-throughput RNA-sequencing were performed with the GAIIx platform (Illumina) and RPKM (reads per kb per million) was used to estimate the gene expression levels. The short reads were aligned to the *P. patens* genome v1.6 extracted from Phytozome (https://phytozome.jgi.doe.gov/pz/portal.html) and counted.

### Comparison to publicly available RNA sequencing data

Due to the limited technical repeats of our RNA sequencing time course, we compared our data to an already published RNA sequencing data set (Shinozawa et al., 2019). The data set compared the expression changes after 12 hours of 10 µM ABA treatment against the mock treatment. These treatments were done for wild-type plants and for *snrk2qko* plants, respectively.

### Clustering and GO term analysis

To identify a gene cluster of co-expressed genes in the ABA time-course RNAseq dataset, the clust program was used (Abu-Jamous & Kelly, 2018). Data normalization was performed by the recommended method for RNA-seq (code 101,3,4 meaning quantile normalization, Log2, and Z-score). Subsequent hierarchical clustering of the clusters identified by clust was performed with ShinyGO software v0.76.3 (Ge et al., 2020) using *P. patens* annotation version v1.6. The parameters used were an FDR cutoff of 0.05, a display of the 10 most significant categories with a minimum pathway size of 2 was chosen.

## Results

### ABA decreases apical stem cell division and growth in an SnRK2-dependent manner

**Figure 1:**
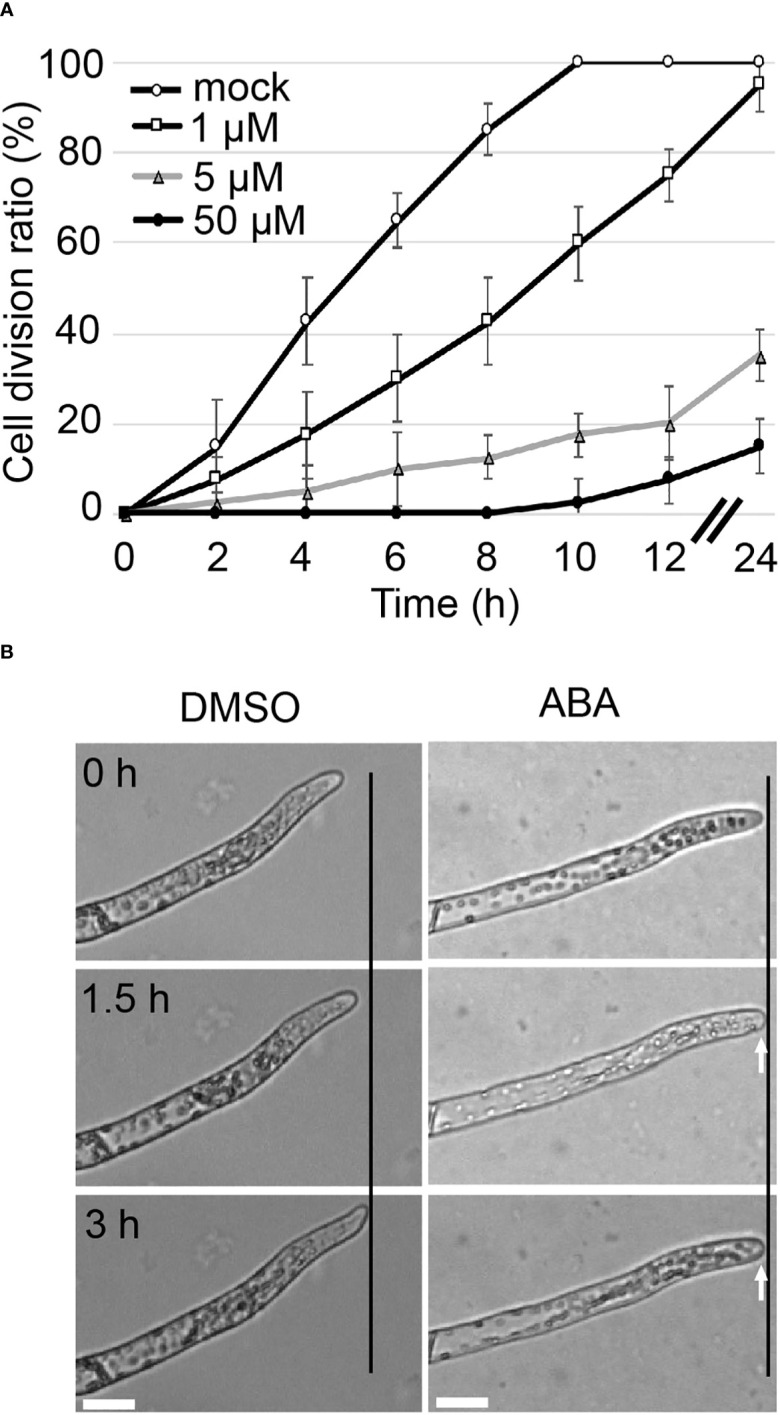
Tip growth is suppressed immediately upon ABA treatment. **(A)** Cell division ratio of protonemal cells in a time course. Protonemal cells grown in glass-bottom dishes for 3 days were subjected to mock or ABA treatments. The cells were observed from 6 h after reagent treatment (t=0). The percentage of apical cells that passed the M phase was determined at different time points. Each data point represents 4 independent experiments with n =10 each. All data are represented as means ± SD. No SD bar was displayed where cell division ratio was at 0 or 100% because all cases showed the 0 or 100% of cell division. **(B)** Time-lapse imaging of tip growth in 0.1% DMSO as a mock treatment (left) and 50 µM ABA treatment (right). 0 h indicates the start of treatment. The apical cell continued to elongate with mock treatment, but with ABA treatment, it stopped elongating after 1.5 hours and its apical apex became round, as indicated by white arrows. Micrographs indicate the respective time course of 1 exemplary cell. Each treatment was carried out with n = 5-8. Black lines indicate the same X-axis position. Scale bar = 20 µm.

Although it has been reported that ABA inhibits growth in a concentration-dependent manner in *P. patens* (Thelander et al., 2005), the direct relationship between ABA and cell division is not well understood. To clarify the relationship between ABA and cell division, apical stem cells of chloronemata were observed over time. The division rate was examined as the percentage of apical cells that had passed the M phase. In mock treatment, all apical cells were dividing at 10 hours after the start of observation, while in 1 µM, 5 µM and 50 µM ABA treatments, only 75%, 20% and 7.5% of the cells, respectively were dividing at 12 hours and only 95%, 35% and 15% of the cells, respectively were dividing at 24 hours (**Figure 1A**). These observations suggest that the apical cell division rate was decreased by ABA in a concentration-dependent manner. To assess the time frame in which apical cell growth is inhibited, the growth of apical cells with mock treatment (DMSO) and with 50 µM ABA treatment was observed (**Figure 1B**). After 1.5 h, apical cells that had been treated with ABA stopped elongating and the shape of the apex of the apical cell changed to a rounder structure compared to that of the same cell at time 0.

**Figure 2:**
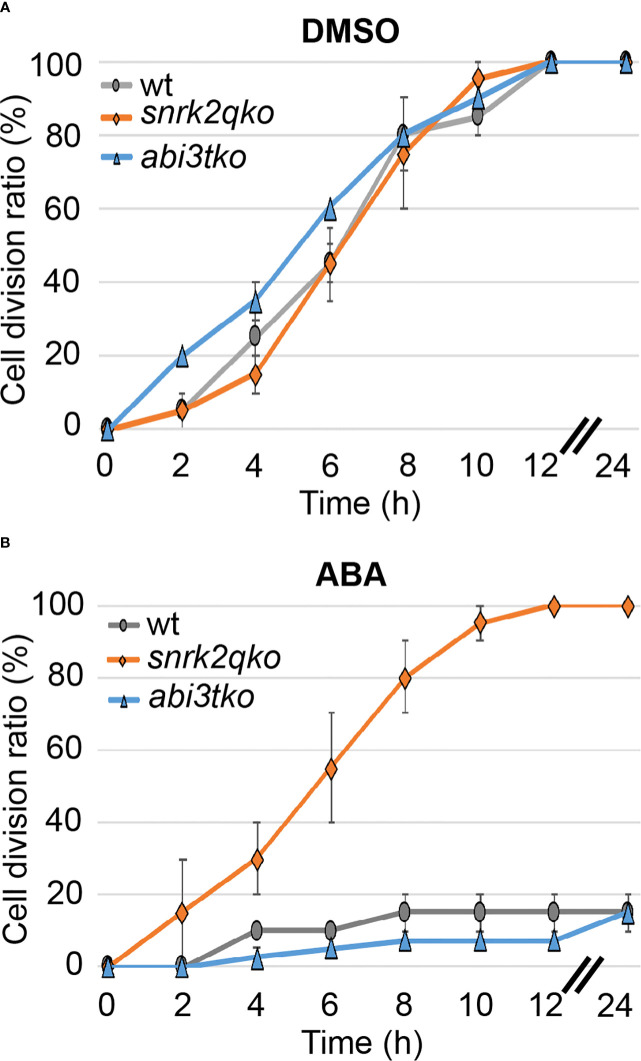
ABA suppresses cell cycle progression *via* SnRK2, not by ABI3. Cell division ratio of protonema apical cells in a time course. WT, *snrk2qko* and *abi3tko* were observed from 1 h after reagent treatment (t=0), and the percentage of apical cells passing the M phase was determined at each time interval. Each point is shown as the mean ± SD of 3 independent experiments with n=10- 20 each. In the 0.1% DMSO treatment **(A)**, all of the cells were divided after 12 hours. In the 50 µM ABA treatment, on the other hand, *snrk2qko* showed a division ratio similar to that of DMSO, but WT and *abi3tko* showed a decrease in the division ratio **(B)**. When the SD value was between 0 and 2.5%, no or little SD bar was seen in such plots.

To determine whether the ABA regulatory kinase SnRK2 and the transcription factor ABI3 that is regulated in a SnRK2-dependent and independent manner (Zhao et al., 2018; Komatsu et al., 2020), are involved in the regulation of cell division, we examined whether the cell division ratio changes in an ABA-dependent manner until 72 hours after the start of ABA treatment. For that purpose, we used a quadruple knockout mutant of snrk2 (*snrk2qko*) and an abi3 triple knockout (*abi3tk*o) that have already been established (Khandelwal et al., 2010; Shinozawa et al., 2019; Tomoi et al., 2020). While no difference from the WT line was observed during mock treatment (**Figure 2A**, DMSO), the *snrk2qko* line showed no decrease in the cell division ratio when treated with 50 µM ABA (**Figure 2B**, ABA). This is unlike WT and the *abi3tko* line, which showed cell division of less than 20% of apical cells that had passed the M phase. This indicates that suppression of cell division and therefore suppression of cell cycle progression by ABA are regulated by SnRK2, not by ABI3. As shown in **Figure S1A**, during ABA treatment for 72 hours, the shape of WT chloronemal cells changed from a rod-like cell shape to a more swollen cell shape with an increasing number but reduced size of chloroplasts. Intriguingly, ABA treatment of WT and *abi3tko* plants did not completely halt the cell division but drastically slowed it down up to 24 hours after the start of ABA treatment. Thereafter, all of the observed apical cells resumed cell division until 72 hours (**Figure S1B**). The results suggest that ABA treatment initially inhibited cell cycle progression, but that cell division resumed due to acclimation. It should be noted that the rate of cell division under the condition of ABA treatment (85% of the WT cells having passed the M phase between 24 and 72 hours, **Figure S1B**) was slower than the division rate under the condition of mock treatment (100% of the WT cells having passed the M phase in 12 hours, **Figure 2A**), indicating that cell cycle progression is differently regulated in the acclimated cells (brood cells) and in the mock and WT cells; i.e., cell cycle progression is most likely delayed in the ABA-acclimated brood cells.

### Apical cell growth inhibition is substantiated through inhibition of asymmetry in cytoskeleton, mitotic spindle and vacuoles

**Figure 3:**
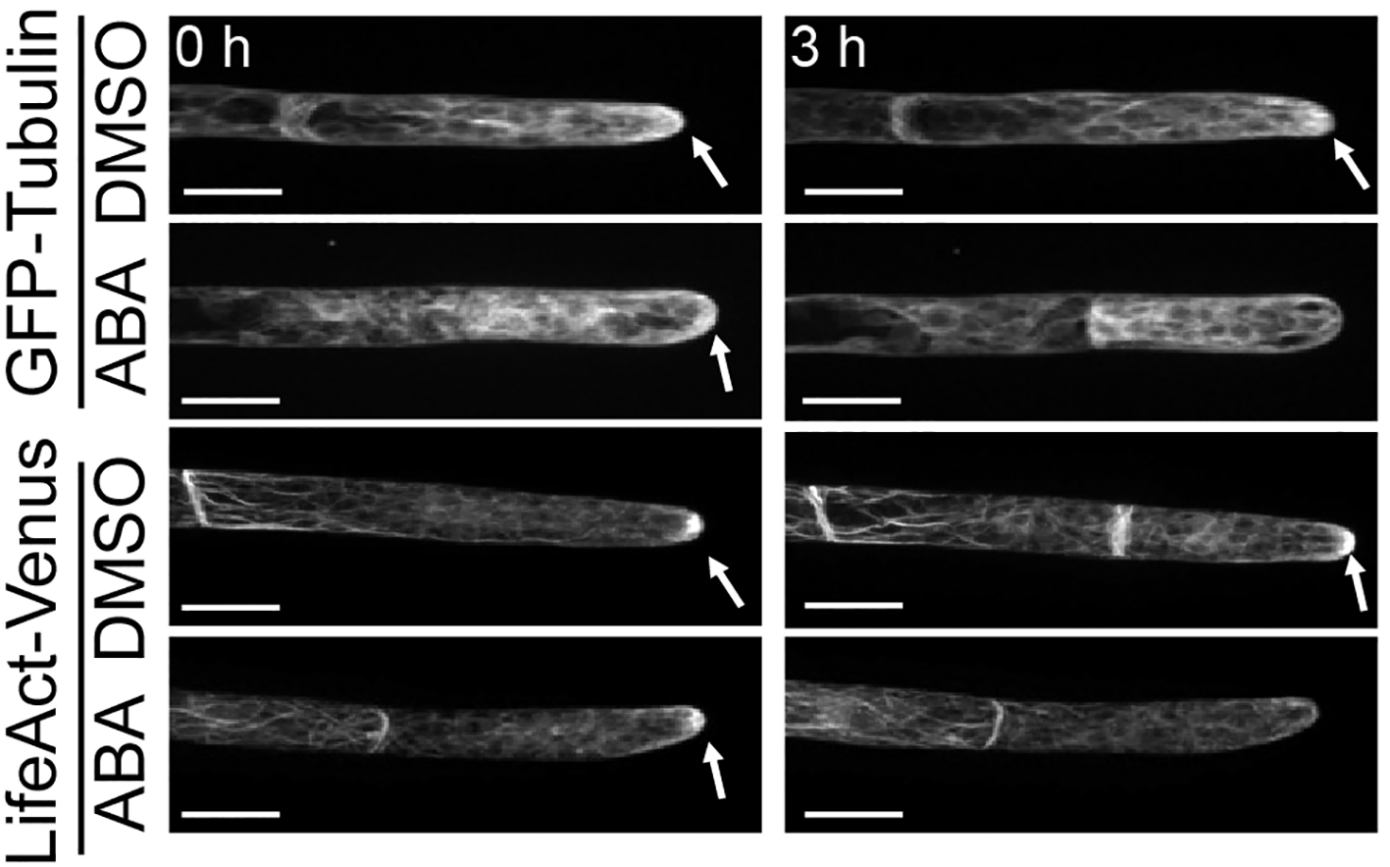
Suppression by ABA of microtubule and actin localization on the apical apex. Fluorescence images of protonema apical cells expressing GFP-Tubulin and LifeAct-Venus that were treated with 0.1% DMSO or 50 µM ABA. Images were taken immediately (0 h, left) and 3 hours (right) after the reagent treatment. White arrows indicate the fluorescence accumulation at the apical apex. After ABA treatment for 3 hours, the fluorescence accumulation on the apical apex disappeared in both cells expressing GFP-Tubulin and cells expressing LifeAct-Venus. 5–8 independent apical cells were observed in each condition with a confocal microscope. Scale bar = 20 µm.

**Figure 4:**
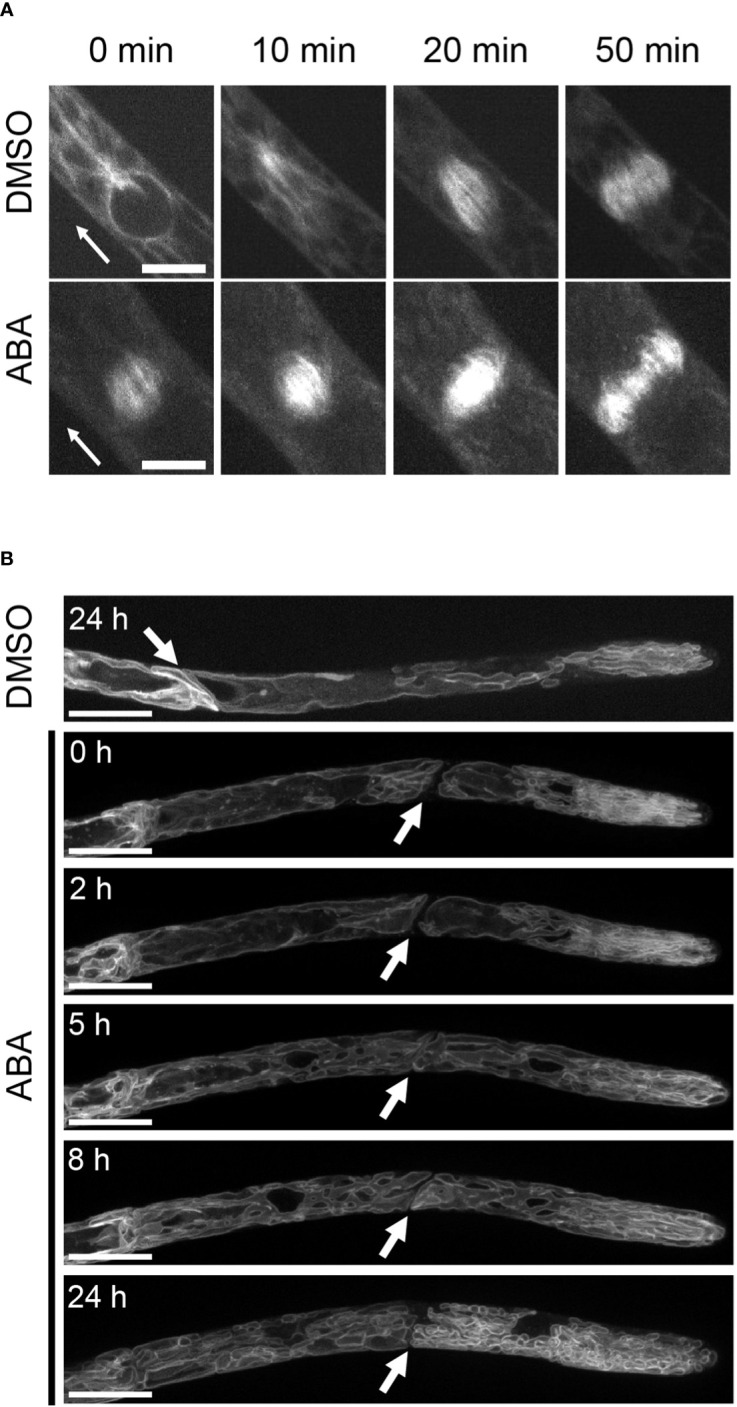
Suppression by ABA of spindle formation and vacuolar asymmetry. **(A)** Time-lapse images of GFP-Tubulin in the cell division zone treated with 0.1% DMSO or 50 µM ABA. In the DMSO treatment, GFP-Tubulin fluorescence accumulated on the apical side (arrowhead direction) in the early stage of spindle formation, and in ABA treatment, the fluorescence polarity disappeared from the early stage of cell division. n = 2-5. Scale bar = 10 µm. **(B)** Fluorescence images of apical cells expressing GFP-AtVam3 that were treated with 0.1% DMSO or 50 µM ABA. The treatment duration is shown in each micrograph. White arrows indicate cross walls between apical cells and subapical cells. In mock and ABA treatments (0 and 2 hr), large vacuoles were observed at the basal side of the apical cell and tubule-like vacuoles were observed at the apical side. After 5 hr of ABA treatment, the vacuoles were broken into smaller pieces. Five independent caulonemal cells were observed. The micrographs represent one exemplary sample, one for DMSO and one for the ABA time course. Scale bar = 20 µm.

Although we proved that the SnRK2 pathway is involved in the ABA-induced suppression of cell cycle progression, the cell physiological cause remains unknown. Since the cytoskeleton plays an important role in the maintenance of asymmetric cell division, we analyzed the distributions of tubulin (GFP-Tubulin) and actin (LifeAct-Venus) in apical cells with and without ABA treatment. In all mock treatments with DMSO and at 0 hours of ABA application, actin and tubulin show a clear preference for apical tip localization as indicated by white arrows in **Figure 3**. After treatment with ABA for 3 hours, no preferential localization of tubulin and actin at the apical tip was observed (**Figure 3**). Since the localization of microtubules and actin at the tip region is an important function of tip growth (Hiwatashi et al., 2014; Wu & Bezanilla, 2018), this indicates that the ABA-induced decrease of apical growth is partially caused by the breakdown of apical tip localization of cytoskeletal components. We also used GFP-Tubulin to observe asymmetric spindle formation, an important step for asymmetric cell division (McCarthy & Goldstein, 2006; Yamada & Goshima, 2017), under the influence of ABA. While a clear spindle-like microtubule asymmetry towards the tip position of the apical cell was observed under the condition of mock treatment, indicating prospindle (prophase spindle) at 0 min and mitotic spindle (metaphase spindle) at 10 min (**Figure 4A**, DMSO), ABA treatment led to symmetric mitotic spindle formation (**Figure 4A**, ABA at 0 min). Thus, ABA breaks the asymmetry of the monopolar spindle so as to form a bipolar spindle in brood cells.

The observed decrease in apical cell growth led us to analyze the vacuole, since an economic way for cell elongation is enlargement of vacuoles (Krüger & Schumacher, 2018). We therefore analyzed the effect of ABA on the shapes of vacuoles in GFP-AtVAM3-overexpressing lines (Oda et al., 2009). While the DMSO mock treatment showed a large vacuole at the basal side of the apical stem cell, tube-like vacuolar structures were observed on the apical side (**Figure 4B** and **Figure S2**). However, this vacuolar asymmetry was gradually lost during the ABA treatment, with remarkable changes after 5 hours. The resulting vacuolar structure after ABA treatment for 24 hours was an evenly distributed tubular or much smaller vacuoles throughout the cell. It should be noted that the large central vacuole in the sub-apical cell was also segmented, giving rise to numerous tubulate or smaller vacuoles (**Figure S2**). Thus, our findings for the cytoskeleton, spindle morphology, and vacuolar size and distribution support the idea that ABA widely attenuated phenomena related to cell polarity, and this may explain the inhibition of apical stem cell growth.

### ABA induces transcriptional changes of gene clusters related to organelles

**Figure 5:**
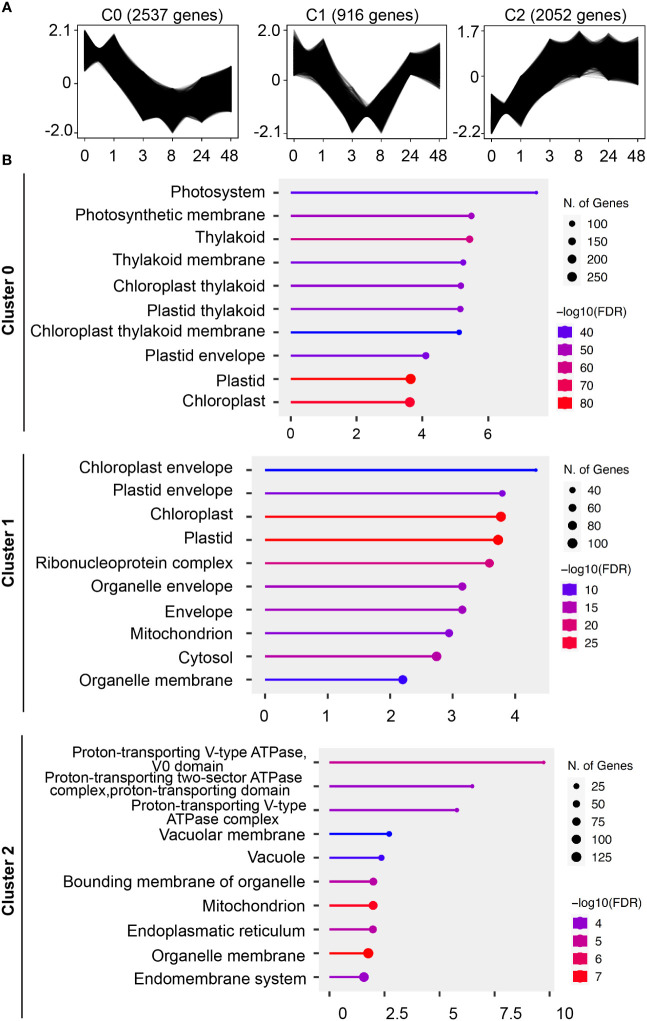
Identification of cellular structures influenced by ABA through co-expression and GO-term analysis. **(A)** Gene profiles of co-expression clusters identified by the clust program in an ABA time-course RNAseq dataset. Protonemal cells grown in BCDAT media were supplemented with 50 µM ABA and samples were taken at 0, 1, 3, 8, 24 and 48 hours after media transfer. The time points are represented on the x-axis, and the y-axis represents the normalized gene expression value. The sub-plot titles indicate the cluster name and the number of genes in the respective cluster. **(B)** Pathway enrichment analysis of the time-course ABA RNAseq dataset. Top significant over-represented pathways revealed in the gene expression clusters identified by clust. Functional enrichment of the 10 most significant categories of cellular components (GO terms). Hierarchical clustering was performed with ShinyGO software v0.76.3 using *P. patens* gene names v1.6. The x-axis indicates the fold enrichment and the y-axis represents the cellular component GO terms. The number of genes is indicated by the dot size and the colors represent the size of the negative log10 of the false discovery rate (FDR).

While we identified cellular changes involved in the tip growth reduction through ABA, we were also expecting more long-lasting effects. We therefore analyzed the transcriptional changes upon ABA treatment in a time course (0, 1, 3, 8, 24, and 48 hours and 5 and 10 days). A co-expression cluster analysis was chosen to reflect larger regulatory groups. When the time points of 5 and 10 days were included, only one cluster mainly consisting of transcription/translational related GO terms was identified (**Figure S3**). Since this regulatory change was only important after several days of treatment, we excluded both of the time points (5 and 10 days) for subsequent analysis. We then identified 3 gene co-expression clusters that show a downregulation, a transient-downregulation, and an upregulation, respectively (**Figure 5A**).

To link the physiological findings to the results of RNAseq analysis, we performed GO term enrichment analysis for the cellular compartment category (**Figure 5B**). Cluster C0 with continuous downregulation shows GO terms related to photosynthetic organelles, which is similar to cluster C1 with transient downregulation and GO terms for photosynthetic and other organelles such as mitochondria. The C2 cluster with a constant upregulation after 1 hour of ABA treatment is mainly associated with GO terms that are regulated to the vacuole and the intracellular membrane system.

### ABA has a limited effect on genes regulating cell polarity

We used the same RNAseq dataset to further analyze the effect on genes regulating cell polarity. We focused on two components, guanine exchange factors (ROPGEFs) and small GTPases (ROPs), which are well known to be important for cell polarity and tip growth. A gene list of all 4 ROPS and 6 ROPGEFs is listed in **Supplemental Table 1**. All 4 ROPs of *P. patens* follow the same expression pattern, with a steady decrease during the first 8 hours followed by a recovery to about half of the original value after two to five days (**Figure S4A**). The behavior of ROPGEFs is more versatile, though the general expression value is lower than that of ROPs (**Figure S4B**). ROPGEF 1 and 2 are expressed at low levels to begin with, whereas the expression levels of ROPGEF 3,4 and 6 drop drastically within the first hour, with a partial recovery in the expression level of ROPGEF 4 after 5 days. The strongest regulation with an adverse pattern was observed for ROPGEF 5, which is shown in a separate graph due to its strong expression (**Figure S4C**). The expression level of ROPGEF 5 increased 3 fold within the first hour, though the expression level returned to its original expression after one day and then gradually decline to nearly zero over a period of 10 days.

### ABA inhibits cell cycle regulators

Unexpectedly, genes that regulate the cell cycle were not included in the gene clusters identified in the ABA time course treatment, although a delay of cell division was observed (**Figure 1A**). We therefore checked the expression pattern of major cell cycle regulator genes in ABA time course treatment, since they represent a major pathway for growth regulation (Ishikawa et al., 2011). The observed genes are based on the homologues of *A. thaliana* cell cycle genes, as listed in **Supplemental Table 1** and indicated in the article by Ishikawa et al. in 2011. A major regulatory step of the cell cycle for the G1 to S phase transition is based on the interaction of D-type cyclin (CYCD) and A-type-cyclin-dependent kinase (CDKA), which form a CDKA/CYCD complex. This complex regulates the E2 promoter binding factor (E2F), retinoblastoma-related (RBR) and dimerization partner (DP) cell cycle regulators, leading to progression of the cell cycle (Inzé & De Veylder, 2006; Oakenfull et al., 2002). In addition, B-type cyclin (CYCB) and B-type-cyclin-dependent kinase (CDKB) are functional in the G2 to M phase transition in *A. thaliana* and *P. patens* (Inzé & De Veylder, 2006; Ishikawa et al., 2011).

**Figure 6:**
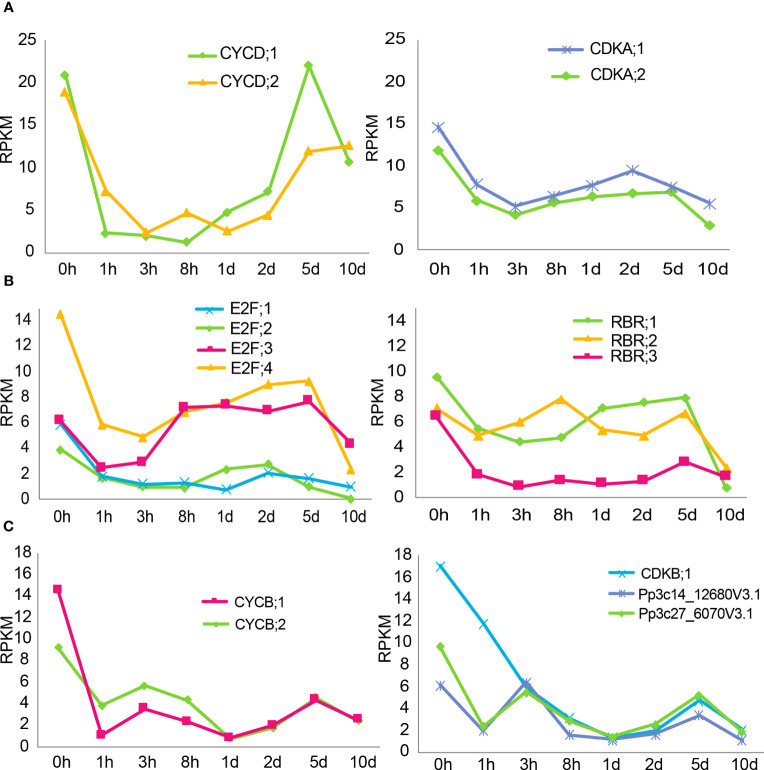
Expression changes of cell cycle genes during ABA treatment. **(A, B)** Gene expression profiles of genes related to the G1 to S phase transition in an ABA time-course RNAseq dataset. **(A, B)** have different y-axes. Protonemal cells grown in BCDAT media were supplemented with 50 µM ABA and samples were taken at 0, 1, 3, 8, 24 and 48 hours and 5 and 10 days after media transfer. The time points are represented on the x-axis, and the y-axis represents the RPKM values. **(C)** Gene expression profiles of genes related to the G2 to M phase transition in an ABA time-course RNAseq dataset. Protonemal cells grown in BCDAT media were supplemented with 50 µM ABA, and samples were taken at 0, 1, 3, 8, 24 and 48 hours and 5 and 10 days after media transfer. The time points are represented on the x-axis, and the y-axis represents the RPKM values.

All of the genes related to the G1 to S phase transition are shown in **Figures 6A, B**. It should be noted that the y-axis in **Figure 6** is different for the panels. The CYCD;1 and CYCD;2 genes show the strongest downregulation after 8 hours and 3 hours, respectively, with a subsequent recovery (**Figure 6A**). The complex partners CDKA;1 and CDKA;2 are less influenced, with an expression level drop to half of the original value after 3 hours. The response of the subsequent complex with E2F and RBR genes is more diverse. All of the E2F genes show an early temporal decrease, while the subsequent recovery depends on the genes corresponding to individual isoforms (**Figure 6B**). Interestingly, after 10 days, the expression values of all E2F genes drop again. The RBR;1 and RBR;2 genes show a more minuscule decrease combined with a sharp decrease at 10 days to new lows. The expression value of RBR;3 drops fast to 1/6^th^ of the value after 3 hours and remains at a low level.

All of the genes related to the G2 to M phase transition are shown in **Figure 6C**. The expression value of CYCB;1 drops to its lowest value after 1 hour, followed by partial recovery. The expression value of CYCB;2, on the other hand, takes 24 hours to reach its lowest value but recovers thereafter. There are seven putative CDKB genes in the genome and the expression levels of four of them were relatively low and barely changed during the time course experiment. In contrast, the expression levels of CDKB;1 and the other two CDKB genes (based on the annotation in phytozome, all listed in **Supplemental Table 1**) dropped in a nearly linear fashion during the first 24 hours, followed by a slight recovery.

## Discussion

### The ABA core component regulates tip growth and cell division in apical stem cells

The core components of ABA signaling with PYR/PYL/RCAR ABA receptors, PP2C phosphatases and SnRK2 lead to the direct phosphorylation of many transcription factors such as ABFs (ABA-responsive element-binding factors) and ABI5 (Furihata et al., 2006). There are also transcription factors such as ABI3 that are essential and promotive factors of the ABA signaling pathway through interaction with ABI5 (Skubacz et al., 2016). Here, we show that the suppression of apical cell growth upon ABA supply is directly mediated by the core component, SnRK2. SnRK2 decreases the cell division rate in a dose-dependent manner since WT plants show an ABA dose-dependent response, while *snrk2qko* under the condition of ABA supply shows a division rate comparable to that of WT under the condition of no ABA supply (**Figure 2**). WT and *abi3tko* showed comparable behavior under all conditions, and the cell division rate was drastically delayed upon supply of 50 µM ABA, though cell division was not completely stopped. Although ABA inhibits protonemal tip growth in a concentration-dependent manner in *P. patens* and within 1.5 hours (Thelander et al., 2005, **Figure 1B**), *snrk2qko* was completely insensitive to tip growth in apical stem cells even at a high concentration of ABA of 50 or 100 µM (Shinozawa et al., 2019; Tomoi et al., 2020). Taken together, our results indicate that SnRK2 modulates tip growth and cell cycle, probably by direct phosphorylation of targets in an ABA dose-dependent manner.

### A shift to symmetric cell division is responsible for the decrease of cell polarity and cell growth

With the major signaling component identified, we focused on identification of the underlying cell physiological changes. Protonemal apical cells of *P. patens* that function as stem cells due to their asymmetric cell division are tip growing cells. From studies in pollen tubes, a model for tip growing cells, it is well known that the polar elongation is maintained through a polarized secretion that is based on vesicle transport. The cytoskeleton, specifically actin filaments are important for site-directed targeting of vesicles and therefore maintain polar growth (Yong et al., 2008; Wang et al., 2013). This is in good agreement with our data and previous observations in *P. patens* protonemal cells, indicating polar localization of actin and tubulin at the apical tip (Hiwatashi et al., 2008; Vidali et al., 2010; Rounds & Bezanilla, 2013). However, this asymmetry is lost within 3 hours after ABA supply (**Figure 3**), which coincides with the growth arrest. This indicates that the polar distribution of actin and tubulin in the apical stem cells is involved in the maintenance of polar growth and that ABA disrupts the accumulation of these cytoskeletal clusters at the tip region and inhibits tip growth.

Another factor that is important for cell growth, especially during elongation, is the plant vacuole (Dünser et al., 2019). Vacuoles are a means for responding to the environment as shown in a review (Krüger & Schumacher, 2018). The two main important things are the energetically favorable expansion of the vacuolar volume, allowing an affordable growth, and the ability to switch rapidly to cell expansion when a vacuolar network is given. Both points are represented in our data. When no ABA is supplied, the basal side of the apical cell has a large vacuole, reducing the cost of cell elongation, while the apical side of the apical cell shows tube-like vacuolar structures. However, upon ABA supply, this asymmetry is lost, and tube-like vacuolar structures and smaller vacuoles are observed throughout the cell (**Figure 4B** and **Figure S2**). Since this coincides with a decrease in growth, it indicates that indeed the large vacuole helps to maintain cell growth, while smaller vacuole structures limit cell growth.

Another cell physiological observation is the observation of monopolar spindle formation. Unlike the majority of plant cells that form a bipolar spindle during cell division (Lloyd & Chan, 2005), protonemal apical cells form a monopolar spindle, which is likely important for asymmetrically distributing cellular components and performing proper asymmetric cell division in protonemal stem cells (Yamada and Goshima, 2017). Indeed, the asymmetric distribution of monopolar spindles was disturbed by ABA application, indicating a loss of spindle formation polarity.

Together, these observations, including the observations of the loss of asymmetry in the distribution of actin, tubulin, the vacuole and spindle structure, indicate that the intracellular asymmetry in the apical stem cell was lost under the condition of ABA supply. We propose here that the intracellular asymmetry is an important driver for fast elongation in tip growing stem cells. Taken together, the results indicate that the tip growth is maintained by apical localization of actin and tubulin, which are likely involved in vesicular delivery. The enlarged vacuole at the basal side of the apical cell decreases the cost for expansion, and the tube-like vacuolar structure at the apical side increases the occupancy ratio of the cytosol. Upon asymmetric cell division, the apical area will therefore contain a larger cytosolic volume in relation to the vacuole, and it has enough vacuolar membranes to drive fast expansion, based on the tube-like vacuolar structure. This is also important since the vacuolar volume needs to be small for cell division, allowing accommodation of the volume of the phragmoplast array and associated cell plate-forming structures (Seguí-Simarro & Staehelin, 2006). The ABA treatment is therefore responsible for the loss of intracellular asymmetry and as a consequence the shift to symmetrical cell growth and symmetrical cell division. Loss of intracellular asymmetry is well aligned with cell morphological change from a rod-like shape in protonemal cells to a more swollen, round cell shape (**Figure S1A**), suggesting that ABA is involved in the shift of anisotropic tip growth to isotropic diffusive growth.

### Cell organelle reorganization plays one of the major roles in the ABA-mediated abiotic stress response

To substantiate our cell physiological observations, we continued with a GO term enrichment analysis for the cellular component category in our ABA time-course RNAseq dataset. Interestingly, two out of three co-expression clusters, the ones that are continuously and temporally downregulated (clusters C0 and C1), are related to photosynthetic organelle GO terms. Considering the known responses of plastids to abiotic stress or ABA treatment in *P. patens* (Nagao et al., 2005; Do et al., 2020), we deal here likely with two different phenomena. The constant downregulated cluster might be an indication for a protection mechanism of the photosynthetic system. The apical growth of *P. patens* protonema decreases upon ABA supply and finally brood cells are formed. The brood cells are stress resistant cells but seem to be physiologically quite inactive based on the slower cell division rate. Under these conditions, downregulated photosynthesis would make sense. A possible mechanism would be the switch to cyclic electron flow, which relies on photosystem I. Cyclic electron flow is known to be involved in the drastic modulation of the electron transport chain in stress adaption (Öquist & Huner, 2003). However, since there are large differences between different plant species, a more detailed analysis is necessary to clarify it (Suorsa, 2015). Second, the downregulation of cluster 1 is temporal, indicating a short-term adaption that allows a switch from fast growth to stress resistance. Considering the stress adaptation component, a possible explanation is the adjustment of osmotic pressure as the stress resistance mechanism. Indeed, such a mechanism was suggested to be responsible for ABA-induced freezing tolerance (Nagao et al., 2005). The authors described a volume reduction in plastids caused by the degradation of starch. This degradation of starch led to an accumulation of sucrose in the cytosol, increasing the tolerance against freezing. Since we did not analyze plastids in our experiments and there is an inconsistent report that starch formation was increased after exogenous ABA supply (Arif et al., 2019), such a regulation needs to be further explored.

The 3^rd^ cluster, C2, which is continuously upregulated under the condition of ABA supply, is related to the vacuole and the endomembrane system. As in our ABA treatment, comparable segmented vacuoles were reported upon ABA-mediated freezing tolerance (Nagao et al., 2005). Though energetically these vacuoles are not favorable for cell expansion, the resistance against any form of osmotic stress is increased since the vacuole cannot easily burst anymore. With the volume loss of the large vacuole and the tubulate and/or small segmented vacuole formation, it is necessary to synthesize more tonoplast. A large tonoplast surface area in relation to the vacuolar volume would allow for rapid growth, when the external conditions are more favorable (Krüger & Schumacher, 2018). Taken together, the results indicate that changes in the chloroplast and the vacuole might lead to extended protection of the photosynthesis apparatus and preparation for future growth, respectively, when abiotic stress is relieved.

### ABA affects genes regulating cell polarity and cell cycle progression

With a clear disturbance of polar growth, we analyzed some known regulators of tip growth and cell polarity. The 4 ROP genes of *P. patens* are very similar in structure (Eklund et al., 2010; Ito et al., 2014), and the observed responses to extended ABA treatment are quite similar (**Figure S4**). The general decrease of the expression in a time frame similar to that of the tip growth inhibition is in agreement with the results of a previous study showing a correlation between reduced expression, in this case caused by RNA interference, and reduced tip growth through suppressed actin-filament dynamics (Burkart et al., 2015). Guanine exchange factors, ROPGEFs, are such regulators that switch GDP for GTP on ROP, leading to its activation (Bascom et al., 2019). Out of the 6 ROPGEFs, two had general low expression levels and three were drastically downregulated within the first hour, indicating indeed a lower activity of ROP proteins. ROPGEF 5, however, showed a drastic upregulation with a slow but steady decrease in its expression level over the next 10 days. It is therefore unlikely that ROPGEF 5 is important for the initial inhibition of tip growth, though it should be analyzed in the future.

To analyze the persistent changes during ABA treatment, we focused on major cell cycle genes. Interestingly, all CYCD and CYCB genes, major regulators of the G1 to S phase and G2 to M phase, respectively, were strongly downregulated within the first one to three hours. Intriguingly, while the CYCB expression remained low, CYCD expression recovered drastically to half or even more than the original values in the long-term treatments at 5 days. This indicates that CYCD or the CYCD/CDKA complex might be important for slower cell cycle progression, probably acclimated cell cycle progression observed in brood cells (**Figure S1B**), or for reentry into the cell cycle once the stress or ABA treatment stops. The differential expression patterns of the E2F and RBR genes might reflect a functional diversification in the subsequent regulation of S-phase genes, as reported in *A. thaliana* (Inzé & De Veylder, 2006).

CDK binds to specific cyclins to form the CDK/Cyclin complex, which is then activated for cell cycle progression. Regulation of cyclin gene transcription and protein degradation at a particular cell cycle stage is essential for activating this complex. The minor expression changes of CDKA compared to those of cyclins is consistent with this general concept (Morgan, 2003). In contrast to CDKA, CDKB is slowly but drastically downregulated in the first 24 hours (**Figure 6C**). This indicates that CDKB might play a more significant role in the initial response or cell fate regulation upon stress or ABA treatment. It might therefore be interesting in the future to analyze the CDKB function in the cell fate transition and its reversal when the stress is released.

To confirm the validity of our RNAseq results, we compared the trends of our time course analysis to an already published data set that corresponds to the expression changes after 12 hours of 10 µM ABA treatment (Shinozawa et al., 2019). All genes analyzed in our data set showed the same trends as in the previously published data set, with the majority of genes decreasing after 12 hours of ABA treatment in wild type, and some showing little or no change (**Supplemental Table 1**). Due to a low read number, 3 proposed CDKB genes could not be confirmed in the published data set. Additionally, a *snrk2qko* line was used to measure the expression changes after a 12 hours treatment of 10 µM ABA. This data set showed that the ABA-dependent suppression on gene expression related to cell cycle and cell polarity is drastically mitigated in the *snrk2qko* line (**Supplemental Table 1**), supporting our finding that ABA decreases apical stem cell division through these genes in an SnRK2-dependent manner. Notably, when the gene alteration under the ABA treatment in wild type is compared to the *snrk2qko* line, the genes CDKB;1, CYCD;1, CYCD;2, CYCB;1, CYCB;2 and ROPGEF 4 showed a strong downregulation in wild type, whereas no such downregulation was detected in the *snrk2qko* line (Shinozawa et al., 2019, **Supplemental Table 1**). This suggests that these 6 genes involved in the cell cycle and the cell polarity may be particularly important targets of the SnRK2 kinase.

Taken together, our results confirmed that ABA signaling is indeed a two-edged sword. The cell fate conversion of apical cells, a stem cell type of *P. patens* protonema to brood cells, resistant but slowly growing stem cells, is mediated by the core component of the ABA signaling pathway. A cell fate switch of stem cells from asymmetric to symmetric division is known in mammals and is a potential interesting target for future research. Cell polarity loss is implicated on a cell physiological level by a change from asymmetric to symmetric distribution of actin, tubulin and spindle formation, and some ROPs and ROPGEFs would be potent targets for ABA signaling. The growth arrest and acquirement of stress resistance increase are furthermore substantiated by changes in the vacuole and plastids, leading to a decrease in turgor pressure and an increase in osmotic components, though further study is needed to determine the molecular mechanism by which the tradeoff is regulated. It would also be interesting to analyze cell cycle changes and their connection to the organelle organization after stress release since brood cells give rise to normal apical stem cells, resuming their growth. Finally, we propose that a further understanding of the process of transition between two different types of stem cells in the moss would enable elucidation of the detailed molecular mechanism of the tradeoff between growth and stress response and elucidation of how stress-resistant stem cells are generated from normal stem cells and vice versa at the cellular and molecular levels.

## Data availability statement

The RNA-seq dataset presented in the study are deposited in the DNA Data Bank of Japan (DDBJ) under the accession number of DRA (DDBJ Sequence Read Archive) 017298.

## Author contributions

MB: Writing – original draft, Investigation, Writing – review & editing. CJ: Investigation, Writing – original draft, Writing – review & editing. NN: Investigation, Writing – review & editing. KN: Investigation, Writing – review & editing. SS: Data curation, Methodology, Writing – review & editing. YS: Data curation, Methodology, Writing – review & editing. TF: Conceptualization, Funding acquisition, Supervision, Writing – original draft, Writing – review & editing.
