# Supplementary material for: ABA signaling converts stem cell fate by substantiating a tradeoff between cell polarity, growth and cell cycle progression and abiotic stress responses in the moss Physcomitrium patens
Source: Front Plant Sci. 2023 Nov 29;14:1303195. doi: 10.3389/fpls.2023.1303195 (PMC10716362; doi:10.3389/fpls.2023.1303195)
Supplement: Supplementary file 1 [file DataSheet_1.pdf]

Supplementary Material

Supplementary Figures and Table

ABA signaling converts stem cell fate by substantiating a tradeoff between cell polarity, growth and cell cycle progression and abiotic stress responses in the moss *Physcomitrium patens*

Marcel Pascal Beier[1,2†], Chiyo Jinno[3†], Natsumi Noda[1], Kohei Nakamura[3], Sumio Sugano[4] Yutaka Suzuki[4], Tomomichi Fujita[1 *]

[†]These authors contributed equally to this work and share first authorship

*** Correspondence:** Tomomichi Fujita: tfujita@sci.hokudai.ac.jp

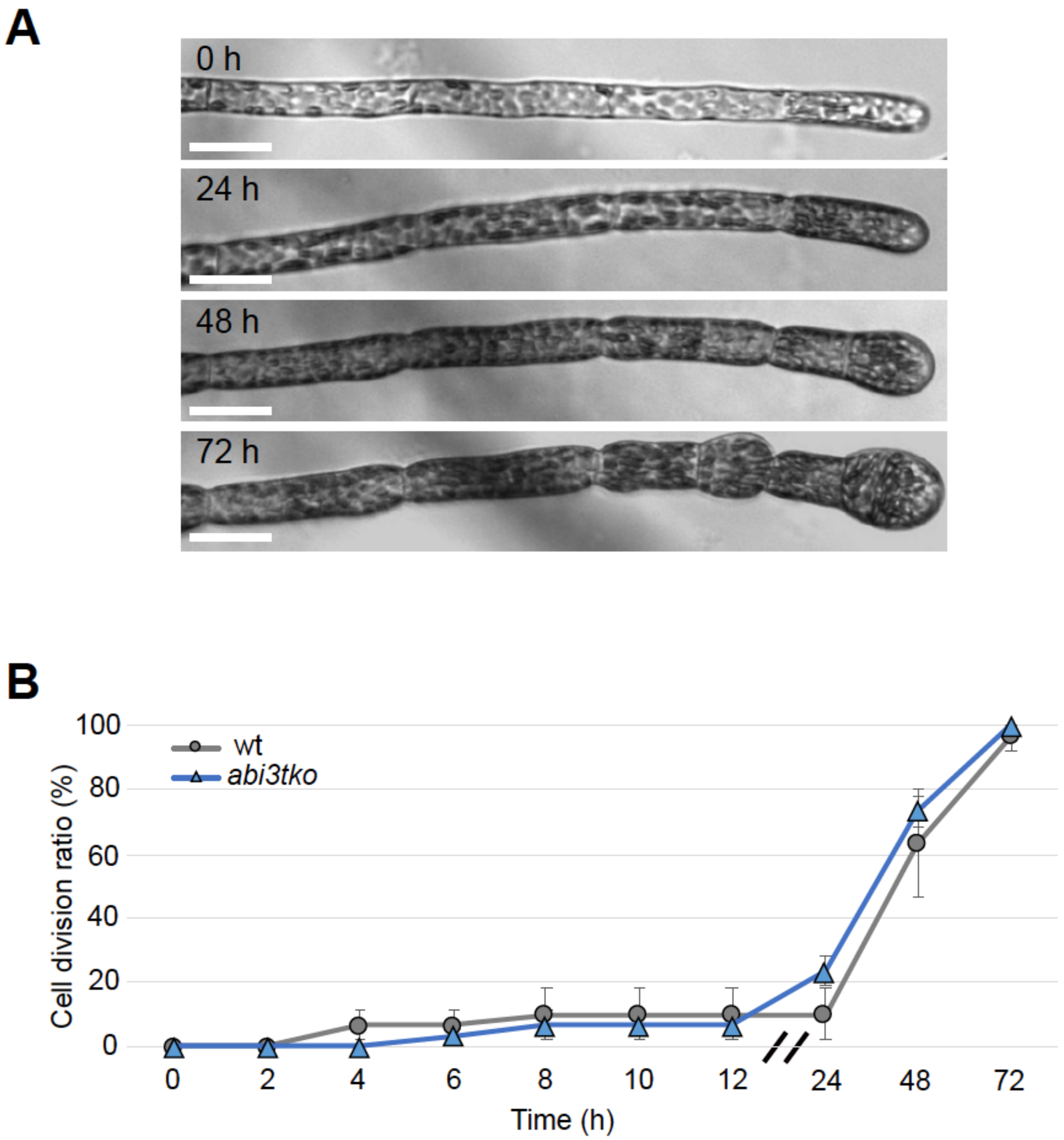

Supplemental Figure 1: Cell division is delayed but not completely suppressed by ABA supply in WT and *abi3tko* plants. (A) Morphological change of protonemal cells during 50 µM ABA treatment for 72 hours.The treatment duration is shown in each micrograph. Cells were observed from 1 h after reagent treatment (t=0). Scale bar= 20 µm. **(B)** Cell division ratio of protonema apical cells in a time course. WT and *abi3tko* were observed from 1 h after reagent treatment (t=0), and the percentage of apical cells passing the M phase was determined at each time interval. Each point is shown as the mean ± SD of 3 independent experiments with n=10- 20 each. In the 50 µM ABA treatment for which results are shown in Figure 2, WT and *abi3tko* showed a drastic reduction in the division frequency during a period of 24 hours. However, when the time course was increased to 48 and 72 hours, WT and *abi3tko* both reached 100% cell division. This indicates that although the cell division was delayed, the cells did not stop dividing.

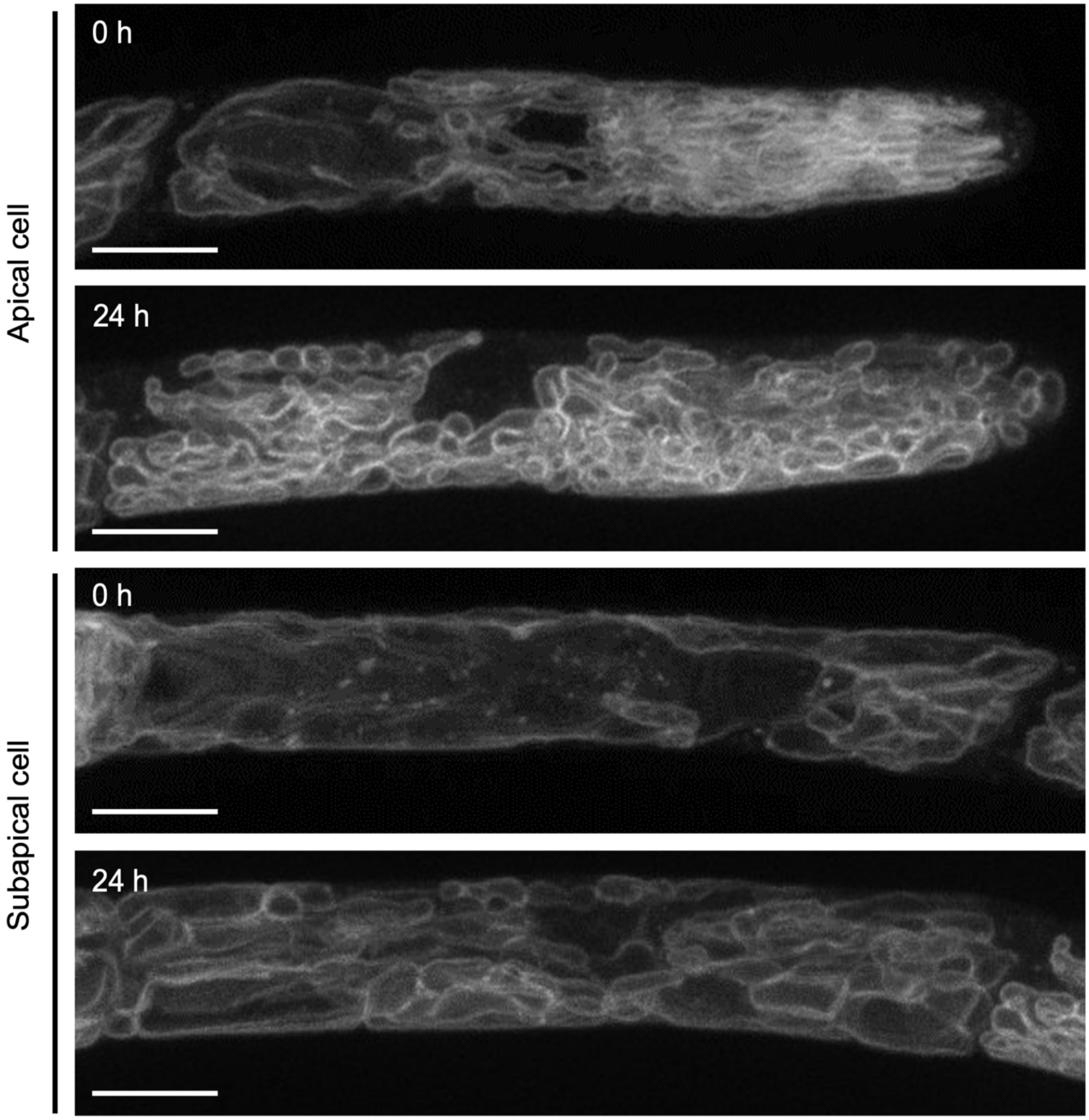

Supplemental Figure 2: Magnified images of apical stem cells and subapical cells in Figure 4. The images show that the large vacuoles in apical stem cells and subapical cells were segmented by ABA treatment to form numerous tubular or small globular vacuoles.

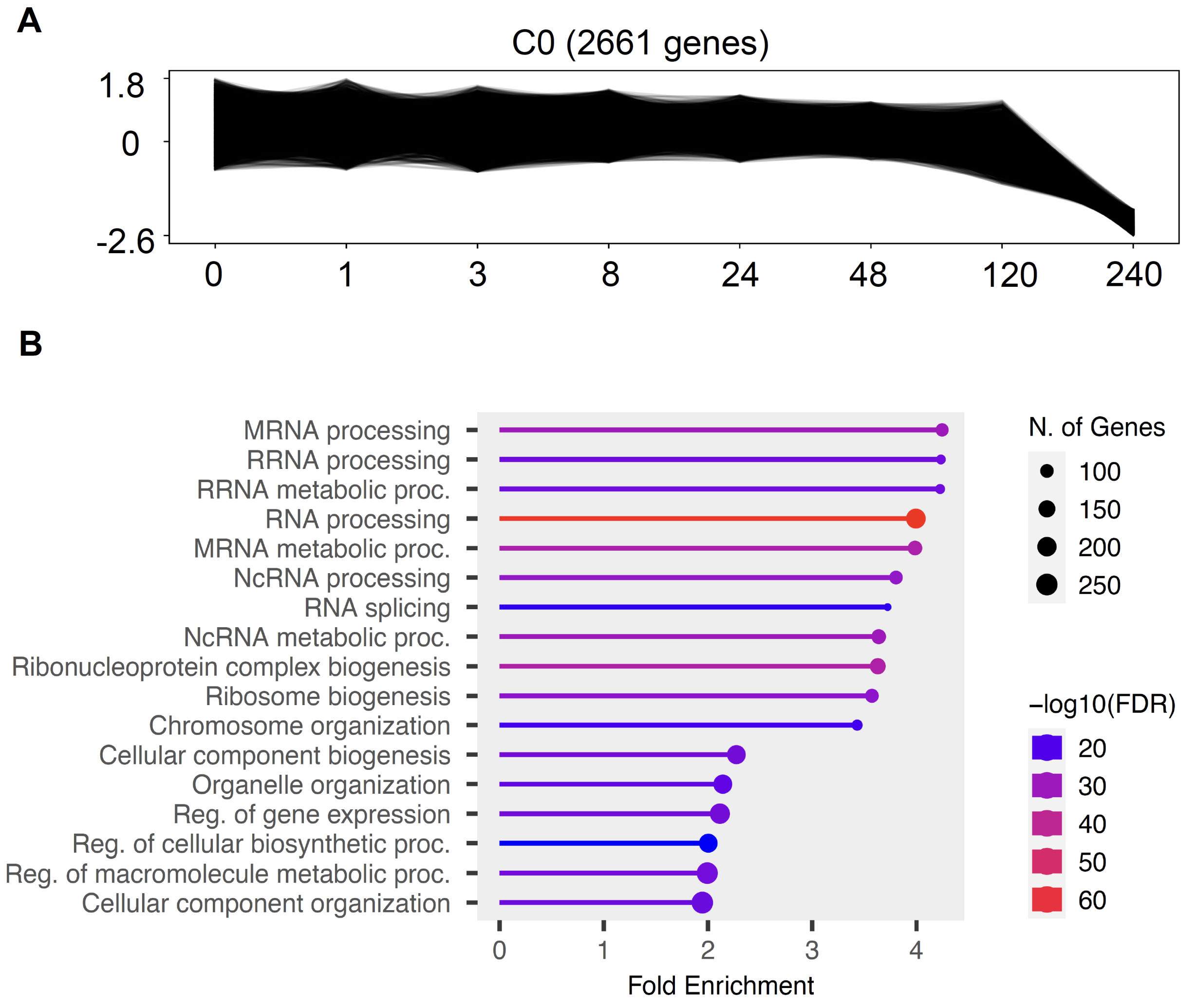

Supplemental Figure 3: Identification of cellular structures influenced by ABA through co-expression and GO-term analysis. (A) Gene profiles of co-expression clusters identified by the clust program in an ABA time-course RNAseq dataset. Protonemal cells grown in BCDAT media were supplemented with 50 µM ABA and samples were taken after 0, 1, 3, 8, 24, 48, 120 and 240 hours after media transfer. The time points are represented on the x-axis, and the y-axis represents the normalized gene expression value. The sub-plot titles indicate the cluster name and the number of genes in the cluster. **(B)** Pathway enrichment analysis of the time-course ABA RNAseq dataset. Top significant over-represented pathways revealed in the gene expression clusters identified by clust. Functional enrichment of the 17 most significant categories of cellular components (GO terms). Hierarchical clustering was performed with ShinyGO software v0.76.3 using *P. patens* gene names v1.6.The x-axis indicates the fold enrichment and the y-axis represents the cellular component GO terms. The number of genes is indicated by the dot size and the colors represent the size of the negative log10 of the false discovery rate (FDR).

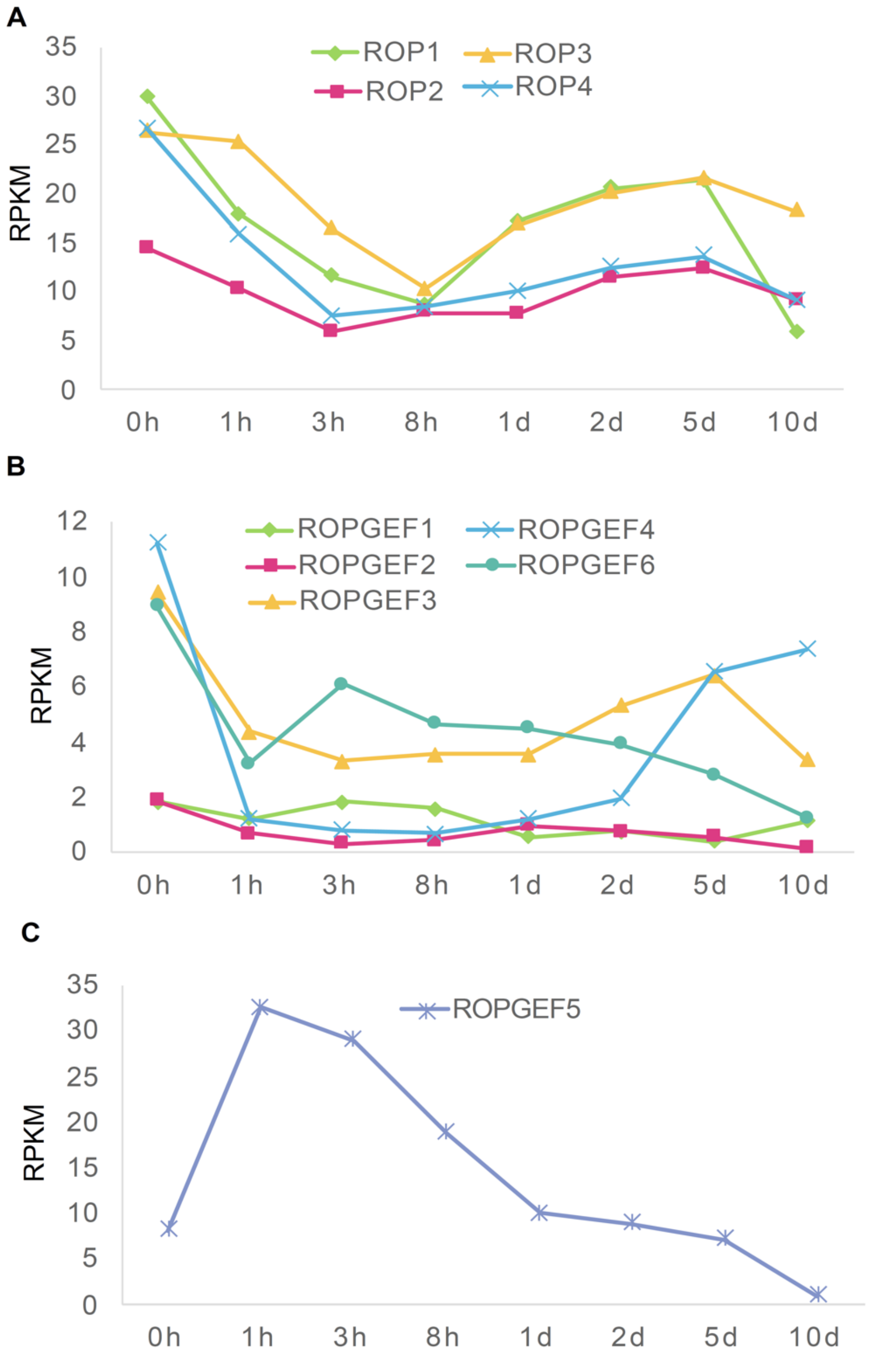

Supplemental Figure 4: Expression changes of cell polarity genes during ABA treatment. Gene expression profiles of genes related to cell polarity in an ABA time-course RNAseq dataset. Protonemal cells grown in BCDAT media were supplemented with 50 µM ABA and samples were taken at 0, 1, 3, 8, 24 and 48 hours and 5 and 10 days after media transfer. The time points are represented on the x-axis, and the y-axis represents the RPKM values. It should be noted that the y-axis scale is different between subfigures.

Gene identification of cell cycle and polarity related genes

Gene and gene identifiers for genes related to the cell cycle and polarity related genes are listed.

All data represneting the fold change for the genees in WT plants or snrk2qko plants are from the Supplementart Data 1 (Shinozawa et al. 2019).

In brief, the fold changes of the genes was determined after 12 hours of 10 μM ABA treatment compared to a 12 hour mock treatment.

**Genes related to cell cycle:**			**Fold change in Wt plants**	**Fold change in *snrk2qko* plants**
**Gene names**	**gene_idv3.3**	**gene_idv1.6**	**ABA 12H / Control 12 H**	**ABA 12H / Control 12 H**
**CDKA;1**	Pp3c3_15290V3.1	Pp1s25_198V6.1	-1.310554001	-1.026112645
**CDKA;2**	Pp3c2_18480V3.1	Pp1s281_21V6.1;Pp1s281_23V6.1	-1.015613885	1.28704733
**CDKB;1**	Pp3c16_3910V3.2	Pp1s127_92V6.1	-22.75341285	1.924968042
**CYCD;1**	Pp3c15_17470V3.1	Pp1s359_22V6.1	-14.65138486	1.727378273
**CYCD;2**	Pp3c9_8300V3.1	Pp1s24_216V6.1	-7.634009333	1.266559453
**CYCB;1**	Pp3c15_21520V3.2	Pp1s271_10V6.1	-29.3986898	1.805538713
**CYCB;2**	Pp3c9_18910V3.1	Pp1s89_130V6.1	-6.289512797	1.816211818
**E2F;1**	Pp3c11_6020V3.1	Pp1s364_42V6.1	-1.810241036	1.023458169
**E2F;2**	Pp3c7_25430V3.1	Pp1s97_96V6.1	-1.204411956	-1.039284579
**E2F;3**	Pp3c2_30010V3.1	Pp1s22_60V6.1	2.256615694	1.173192257
**E2F;4**	Pp3c1_6880V3.1	Pp1s38_356V6.1;Pp1s38_357V6.1	-1.198971061	1.058536081
**RBR;1**	Pp3c19_9596V3.1	Pp1s174_126V6.1;Pp1s174_127V6.1	-1.082999499	1.106868726
**RBR;2**	Pp3c9_2440V3.1	Pp1s90_238V6.1	1.260996429	1.429369459
**RBR;3**	Pp3c15_2800V3.1	Pp1s124_108V6.1	-3.300659081	1.50863688
**CDKB (proposed)**	Pp3c1_31370V3.1	Pp1s397_37V6.1	2.438597952	1.41680382
**CDKB (proposed)**	Pp3c11_10380V3.1	Pp1s61_200V6.1	1.823204918	1.540625701
**CDKB (proposed)**	Pp3c14_12680V3.1	Pp1s36_177V6.1;Pp1s36_178V6.1	-3.942114644	1.266442803
**CDKB (proposed)**	Pp3c27_6070V3.1	Pp1s214_1V6.1	N/A	N/A
**CDKB (proposed)**	Pp3c1_5080V3.1	Pp1s45_169V6.1	N/A	N/A
**CDKB (proposed)**	Pp3c7_18430V3.1	Pp1s2_590V6.1	N/A	N/A

**Genes related to cell polarity:**			**Fold change in Wt plants**	**Fold change in *snrk2qko* plants**
**Gene names**	**gene_idv3.3**	**gene_idv1.6**	**ABA 12H / Control 12 H**	**ABA 12H / Control 12 H**
**ROPGEF1**	Pp3c2_4460V3.1	Pp1s7_228V6.1	1.14086592	3.367273291
**ROPGEF2**	Pp3c10_9910V3.1	Pp1s58_274V6.1	-1.10783379	2.059232971
**ROPGEF3**	Pp3c1_20V3.1	Pp1s283_2V6.1	-1.921817039	-1.020223529
**ROPGEF4**	Pp3c2_28420V3.1	Pp1s22_225V6.1	-13.20389236	1.183352892
**ROPGEF5**	Pp3c14_22480V3.1	Pp1s34_404V6.1	1.045115805	-1.233419974
**ROPGEF6**	Pp3c1_36410V3.1	Pp1s28_109V6.1	1.617722271	-1.136147761
**ROP1**	Pp3c14_4310V3.1	Pp1s126_126V6.1	-1.491891353	-1.052590506
**ROP2**	Pp3c2_20700v3.1	Pp1s165_55V6.1	-1.345544306	1.076934165
**ROP3**	Pp3c1_21550V3.1	Pp1s257_74V6.1	-2.376565934	-1.197413284
**ROP4**	Pp3c10_4950V3.1	Pp1s51_114V6.1	-1.756422669	-1.013686755

Supplemental Table 1: Gene identification of cell cycle and polarity-related genes. Gene, gene identifiers and the fold changes for genes related to the cell cycle and polarity-related genes are listed.
